# The Wild Plants from the Family Asteraceae That Are Traditionally Used for Food in Sicily and Bulgaria and Their Health Benefits

**DOI:** 10.3390/foods15060988

**Published:** 2026-03-11

**Authors:** Ekaterina Kozuharova, Giuseppe Antonio Malfa, Rosaria Acquaviva, Vivienne Spadaro, Iliana Ionkova, Giancarlo Statti, Francesco M. Raimondo

**Affiliations:** 1 Department of Pharmacognosy, Faculty of Pharmacy, Medical University-Sofia, 1000 Sofia, Bulgaria; ionkova@pharmfac.nat.bg; 2 Department of Drug and Health Sciences, University of Catania, Viale A. Doria 6, 95125 Catania, Italy; gmalfa@unict.it (G.A.M.); racquavi@unict.it (R.A.); 3 Research Centre on Nutraceuticals and Health Products (CERNUT), University of Catania, Viale A. Doria 6, 95125 Catania, Italy; 4 Department of Biological, Chemical and Pharmaceutical Sciences and Technologies, Section of Botany, Anthropology and Zoology, University of Palermo, Via Archirafi 38, 90123 Palermo, Italy; 5 Department of Pharmacy, Health Sciences and Nutrition, University of Calabria, Via P. Bucci, 87030 Rende, Italy; giancarlo.statti@unical.it; 6 PLANTA/Center for Research, Documentation and Training, Via Serraglio Vecchio 28, 90123 Palermo, Italy; cescoraimondo@gmail.com

**Keywords:** native flora, edible herbs, ethnobotany, health-promoting effects, valorization, biodiversity

## Abstract

This study examines 36 wild Asteraceae species that are traditionally used as food in Sicily and Bulgaria, highlighting their ethnobotanical, nutritional, and pharmacological relevance. Some taxa, such as *Cichorium intybus*, *Silybum marianum*, *Artemisia vulgaris*, *Taraxacum officinale*, and *Tussilago farfara*, are integral to the Mediterranean and Balkan diets, enhancing nutrition through their fiber, minerals, and bioactive compounds. This ethnobotanical survey revealed a clear geographic pattern in species usage: 13 species were found to be consumed solely in Bulgaria, 18 solely in Sicily, and five species in both regions. The distribution highlights the existence of shared culinary traditions that have been distinctly adapted to the unique ecological conditions present in each locale. The main metabolite classes identified include flavonoids, phenolic acids, lignans, and sesquiterpene lactones, all of which are associated with antioxidant, anti-inflammatory, hypolipidemic, and anticancer properties. Specific taxa within the investigated group were found to contain alkaloids that warrant toxicological attention. Some species within the studied group contain alkaloids that may pose toxicity risks. *T. farfara* is known to accumulate pyrrolizidine alkaloids, which are associated with liver damage and potential genotoxic effects, highlighting the importance of thorough toxicological evaluations before recommending these plants for consumption. This study also demonstrates how common culinary processes, such as boiling and blanching, significantly impact the concentration, stability, and safety profile of these bioactive compounds. Overall, the research supports the promotion of wild Asteraceae species as valuable and sustainable nutritional and nutraceutical resources. This approach aligns with efforts to preserve biodiversity and maintain traditional Mediterranean and Balkan food practices, integrating both ecological and cultural sustainability.

## 1. Introduction

Extensive research has shown that plant-derived foods are a rich source of bioactive compounds with diverse health-promoting properties [1,2]. A broad consumption of plant species contributes substantially to the nutritional adequacy of the human diet. Among dietary patterns, the traditional Mediterranean diet is particularly well recognized for its beneficial effects on health [3,4]. Beyond nutrition, it is regarded as a sustainable system encompassing biodiversity, cultural traditions, and dietary practices [5]. Within this framework, species belonging to the family Asteraceae play a prominent role [6]. (These plants have a long history of domestication and cultivation for food purposes, and they are notable for their valuable secondary metabolites [7]. Ethnobotanical investigations provide important insights into plants traditionally consumed over centuries [8], offering opportunities to identify novel candidates for cultivation or domestication with potential health-promoting effects. Certain plant species members of the Asteraceae family that are of particular interest in this regard are characterized by a restricted distribution, limited to the Mediterranean region or even specifically to Sicily [9]).

The objectives of the present study are twofold: (1) to document wild plant species members of the Asteraceae family that are traditionally used as food in Bulgaria and Sicily, and (2) to assess their bioactive constituents and associated health benefits.

## 2. Materials and Methods

Relevant publications were retrieved from the Google Scholar, Web of Science, and PubMed databases using a combination of search terms, including “Sicily,” “Bulgaria,” “traditional,” “wild,” “food,” “plants,” “ethnobotany,” “medicinal”, “compounds,” “metabolites,” “pharmacological,” “toxic” and “health,” among others. We identified the relevant literature published between 1990 and 2025. All the records were screened for eligibility and evaluated for data quality. A total of 263 publications were excluded for the following reasons: (1) the information was not relevant to the research topic; (2) the ethnobotanical data pertained solely to medicinal uses of Asteraceae plants, not as food; (3) the studies addressed traditional food practices but did not include wild Asteraceae plants; (4) the records referred exclusively to the consumption of cultivated Asteraceae plants; (5) the phytochemical and toxicological reports were not reliable.

## 3. Results and Discussion

### 3.1. Ethnobotanical Data

**Figure 1:**
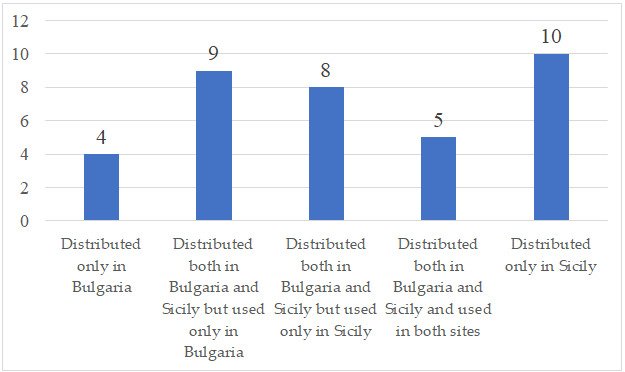
Distribution versus consumption in the study sites.

As a result of the ethnobotanical review, 36 wild taxa of the Asteraceae family are listed as edible (Table 1) and used for food in Bulgaria [8,10,11,12] (and Sicily [13,14,15,16]. Of these listed edible plants, 13 taxa are consumed solely in Bulgaria and 18 taxa solely in Sicily (Table 1, Figure 1). Interestingly, 22 taxa are common for both Bulgaria and Sicily but only five of them are consumed in both regions (Table 1, Figure 1). Furthermore, nine of them are consumed only in Bulgaria and eight only in Sicily (Table 1, Figure 1). *Cynara cardunculus*, commonly known as cardoon or artichoke, is a well-known cultivated vegetable with several varieties [17]. Cultivation of this species has recently expanded, and a multi-use approach has been recommended [18]. Despite its widespread cultivation, *C. cardunculus* is still traditionally gathered from wild populations and consumed in certain countries, including Spain, Morocco, Southern Italy, Sicily, and Cyprus [19].

### 3.2. Bioactive Compounds and Pharmacological Effects

Phenolic compounds are often reported in the listed wild edible plants from the family Asteraceae. These are lignans, flavonoids, and phenolic acids (Table 2).

Many of the consumed traditionally Asteraceae members contain sesquiterpene lactones (Table 2). This topic deserves sharp attention for further research. Sesquiterpene lactones have antioxidant, anticancer, anti-inflammatory, and cardioprotective effects. For example, psilostachyin C (PSC), a sesquiterpenoid lactone identified in *Artemisia vulgaris* (Table 2), is known for its ability to reduce the malignant properties of hepatocellular carcinoma (HCC). The mechanism is explained with the ability of PSC to block the expression of the CREBBP-mediated transcription of GATAD2B and thus it markedly inhibits the proliferation, cell cycle progression, and migration of the tumor cells, while simultaneously inducing apoptosis [20]). However, some sesquiterpene lactones can cause severe toxicity including genotoxicity [21,22].

*Carlina sicula*, *Centaurea cyanus*, *Hypochoeris radicata*, *Onopordum acanthium*, and *Sonchus asper* contain alkaloids. (Table 2). *Tussilago farfara* in particular contains pyrrolizidine alkaloids (senkirkine, senecionine, and saturated otonecine-type tussilagine and isotussilagine) and prenylated indole alkaloids (Table 2).

Essential oils are reported for a few of them, and this seems to be an important element in studies.

**Table 2:** Wild plants from the family Asteraceae traditionally used as food and their major bioactive compounds, therapeutic activity, and toxicity.

Taxon	Major Bioactive Compounds	Therapeutic Activity	Toxicity
*Artemisia vulgaris* L.	Sesquiterpenoid lactones (psilostachyin, psilostachyin C, vulgarin, and artemisinin), flavonoids (kaempferol, quercetin, apigenin, chrysoeriol, rutin, tricin, and vitexin); coumarins (esculin, umbelliferone, and scopoletin); organic acid (quinic acid); phenolic acids (protocatechuic acid glucoside and caffeic acid); sterols (sitosterol and stigmasterol); polyacetylenes; carotenoids; vitamins (ascorbic acid); cyanogenic glycosides (prunasin); and essential oils (1,8-cineole, sabinene, camphor, camphene, caryophyllene oxide, α-thujone, and β -thujone) [23,24,25].	Antioxidant activity: IC_50_: 0.031 mg/mL (DPPH assay) [24]; IC_50_: 4.3 μg/mL (DPPH assay) [25]; IC_50_: 11.4 µg/mL (DPPH assay) [26]; IC_50_: 125 mg/mL (NO assay) [26]. Analgesic activity (in vivo). Effective doses: 200 and 400 mg/kg [25]. Antihypertensive activity (in vivo). Effective doses: 10 mg/mL and 1 mg/mL [23]. Cytotoxic activity: MCF-7 (breast cancer) IC_50_: 190 ng/mL; HeLa (cervical cancer) IC_50_: 284 ng/mL; A7R5 (smooth muscle cells) IC_50_: 382 ng/mL; 293T (transformed kidney cells) IC_50_: 317 ng/mL; A549 (lung carcinoma) IC_50_: 778 ng/mL [26]. Antispasmodic/ antinociceptive activity (in vivo). Effective doses: 500 mg/kg and 1000 mg/kg. Anti-inflammatory/hypolipidemic/hepatoprotective (in vivo). Effective dose: 100–600 mg/kg [26]. Estrogenic, antimicrobial, antiallergic, antimalarial, and gastrointestinal digestion stimulation [26]. Cytotoxicity via mitochondrial dysfunction and caspase activation [27].	Toxic after prolonged use [26].
*Bellis perennis* L.	Flavonoids (apigenin 7-O-β-D-glucoside, apigenin 7-O-β-D-glucuronide, kaempferol, kaempferol 3-O-β-D-glucoside, isorhamnetin 3-O-β-D-galactoside, and quercetin) [28], essential oils (methyl dec-4,6-diynoate and dec-4,6-diynoic acid) [29], and hydroxycinnamates (caffeic acid, rosmarinic acid, chlorogenic acid, neochlorogenic acid, 3,5-dicaffeoylquinic acid, 3,4-dicaffeoylquinic acid, 4,5-dicaffeoylquinic acid, and 3,4-dicaffeoylquinic acid methyl ester) [30].	Antimicrobial, wound healing, nephroprotective, and insulin mimetic effects, as well as an effect on lipid metabolism [31,32]. Anxiolytic/antidepressant-like effects (in vivo). Effective doses: 50, 100, and 150 mg/kg [31]. Antioxidant activity: IC_50_: 168.4 µg/mL (DPPH assay); IC_50_: 74.69 µg/mL (ABTS assay); IC_50_: 78.45 µg/mL (β-Carotene test) [32]. Cytotoxic activity: MCF-7 (breast cancer) IC_50_: 71.6 µg/mL; HepG2 (liver cancer) IC_50_: 73.9 µg/mL [32].	No toxic symptoms [31], but details are still needed [33].
*Carduus nutans* L.	Phenolic compounds such as chlorogenic acid, cryptochlorogenic acid, dicaffeoylquinic acids, kaempferol 3-O-glucoside, kaempferol 3-O-rhamnoside, luteolin, apigenin, kaempferol, diosmetin, tricin, and luteolin O-arabinosyl-glucoside, apigenin O-rhamnosyl-glucoside, and apigenin 7-O-glucoside, and diosmetin acetyl glycosides [34].	Gastrointestinal disorders, liver depurative [35].	Not reported.
*Carlina gummifera* (L.) Less.	Sesquiterpene compounds and acetylenic compound (carlina oxide and 13-methoxy-carlina oxide) [36,37].	100% fidelity level in the local population in Tamalous (north-east of Algeria), specifically to treat a single category of disease (dermatological disorders) [38]) and antioxidant and antifungal activities [36].	Toxicity resides in atractyloside and carboxyatractyloside, two diterpenoid glucosides capable of inhibiting mitochondrial oxidative phosphorylation [39].
*Carlina sicula* Ten.	Terpenoids (sesquiterpene lactones—elemanolides, eudesmanolides, and germacranolides groups), polyphenols, and an alkaloid named siculamide [40].	Assessed is the potential activity on metabolic syndrome; dihydrocnicin and the lignan salicifoliol demonstrate remarkable stimulation of glucose uptake [40].	Not reported.
*Centaurea calcitrapa* L.	Phenolic acids (p-hydroxybenzoic acid, protocatechuic acid, gallic acid, gentisic acid, p-coumaric acid, ferulic acid, caffeic acid, and chlorogenic acid), flavonoids (kaempferol, kaempferol 3-O-glucoside, apigenin, luteolin, chrysoeriol, quercetin 3-O-glucoside, and isorhamnetin 3-O-glucoside) [41], essential oils (β-caryophyllene, 6,10,14-trimethylpentadecan-2-one, and (Z)-β-farnesene, heptanal) [42], sesquiterpene lactones, and lignans [43,44,45].	Antibacterial activity: *S. aureus*; *E. amylovora*; and *X. campestris pv. campestris* MIC: 13–25 µg/mL [41]. Antioxidant activity: IC_50_: 0.84 mg/mL (DPPH assay) [41]; IC_50_: 0.88 mg/mL (ABTS assay) [41]. Cytotoxic activity: MCF-7 (breast cancer) IC_50_: 127.6 µg/mL [44].	All tested concentrations considered as non-toxic [41].
*Centaurea cyanus* L.	Flavonoids (apigenin, luteolin, galangin, kaempferol, catechin, hesperidin, naringenin, quercetin, isorhamnetin, isoquercitrin, naringin, rutin, and pinobanksin); phenolic acids (benzoic, p-aminobenzoic, ferulic, syringic, chlorogenic, salicylic, p-coumaric, vanillic, gallic, and caffeic and sinapinic acids); tocopherols (α-, β-, γ-, and δ-tocopherols); carotenoids (β-carotene and luteolin), lactones, lignans alkaloids, terpenes, and amino acids [46,47].	Anti-inflammatory, skin cleansing, regulating digestion and kidney, gall bladder, liver disorders, and increasing immunity [48].	Photoactive thiophenes, which are potentially toxic [49]. Non-toxic [50].
*Centaurea napifolia* L.	Sesquiterpene lactones (cnicin, 4′-O-acetylcnicin, melitensin, and dehydromelitensin), sesquilignans (lappaol A) [51] and flavonoids (quercetin, hispidulin, cirsimaritin, cirsilineol, and 5-hydroxy-6,7,3′,4′-tetramethoxyflavone) [52]. Essential oil with a low concentration of volatiles and dominating constituents such as palmitic acid and fatty acid methyl esters [53].	Low antimicrobial activity of the essential oil [53].	Not reported.
*Cichorium intybus* L.	Carotenoids (lutein, violaxanthin, antheraxanthin, neoxanthin, and β-carotene), phenolic acids (chlorogenic acid, caffeic acid, and chicoric acid) [54,55]; tannins, saponins [56], sesquiterpene lactones (15-deoxylactucin-8-sulfate, dihydrolactucin-8-sulfate, 11-β,13-dihydrolactucin, lactucin, 8-deoxylactucin, jacquinelin, dihydrolactucopicrin, and lactucopicrin) [57], and inulin [58,59,60].	Traditionally used for loss of appetite, liver disorders, diarrhea, strengthening the prostate and other reproductive organs, pulmonary cancer, hangover, and purification of biliary tract, etc. [61,62]. Antioxidant activity associated with the presence of inulin, caffeic acid derivatives, ferulic acid, caftaric acid, chicoric acid, chlorogenic and isochlorogenic acids, dicaffeoyl tartaric acid, sugars, proteins, hydroxycoumarins, flavonoids, and sesquiterpene lactones [60]. Anti-inflammatory [63], as well as anti-hepatotoxic activity, anti-diabetic, and antimicrobial effects [58], and antiviral properties with potential against SARS-CoV-2 [59]. Cytotoxic activities in vitro and antitumor action in vivo; anticancer potential— *C. intybus* modulates NF-κB and Wnt/β-catenin pathways to inhibit proliferation [27].	The root extract containing sesquiterpene lactones is non-toxic and non-mutagenic even at 1000 mg/kg/day [58].
*Crepis vesicaria* L.	Phenolic compounds with chicoric acid as a major constituent [64]; xanthophylls (violaxanthin, neoxanthin, lutein, zeaxanthin, and β-cryptoxanthin); carotenes (α-carotene, β-carotene, 9-cis-βcarotene, and 13-cis-β-carotene); tocols (in particular about 2–3 mg/100 g of α-tocopherol); thiamine; riboflavin [65]; sesquiterpenes such as 8-deoxylactucin and 11β,13-dihydro-8-deoxylactucin; flavonoids such as luteolin, luteolin 7-*O*-glucoside, and luteolin 4′-*O*-glucoside; phenolic compounds; chlorogenic acid; and 4,4′-dihydroxy-stilbene [57,66].	The traditional use is reported in cases of abdominal colic and anemia, as well as diuretic, hypoglycemic, hypertensive, and laxative effects [63]. Antioxidant activity is reported in DPPH, ABTS, and FRAP assays [65]	Not reported.
*Crepis sancta* (L.) Bornm.	Eudesmane-type sesquiterpenoids (3-oxo-γ-costic acid and its methyl ester); flavonoids (kumatakenin, penduletin, pachypodol, chrysosplenetin, jaceidin, casticin, and quercetin); phenolic acids (3-O-caffeoylquinic acid, 5-O-caffeoylquinic acid, and chicoric and caftaric acids) [67,68,69]).	Antimicrobial, antiviral, antiproliferative, antioxidant, analgesic, vasodilator, and anti-inflammatory activity [68]. Gastroprotective effect (in vivo). Effective doses: 100, 200 mg/kg [68].	Non-toxic [68].
*Crepis leontodontoides* All.	Sesquisterpene lactones (15-deoxylactucin-8-sulfate, dihydrolactucin-8-sulfate, 11β,13-dihydrolactucin, lactucin, 8-deoxylactucin, and jacquinelin) [57,66].	Laxative effect [63,66].	Not reported.
*Cynara cardunculus* L.	Total polyphenols, hydroxycinnamic acids, anthocyanins, chlorophyll, ortho-diphenols, terpenoids, and triterpenoids [70]. Phenolic compounds (chlorogenic acid, p-coumaroylquinic acid, 5-O-feruloylquinic acid, luteolin-7-O-rutinoside, cynaroside, 3,4-dicaffeoylquinic acid, cynarin, luteolin-7-O-malonyl-glucoside, and 4,5-dicaffeoylquinic acid) [71]	Hepatoprotective [72,73], anti-inflammatory [70,74,75,76], and hypoglycemic activity [63]. Anti-angiogenic effects: IC_50_ ≈ 40 µg/embryo (zebrafish) [76]. Antioxidant activity: IC_50_: 20.04 ± 2.52 µg/mL [73].	Absence of toxicity [73].
*Doronicum orientale* Hoffm.	Flavonoids (catechin, kaempferol, rutin, isorhamnetin-3-O-rutinoside, quercetin, hesperidin, and hyperoside and its glucoside derivatives); phenolic acids (p-coumaric, caffeic, ferulic, gallic, chlorogenic, rosmarinic, syringic, dicaffeoylquinic, 4,5-/3,5-/3,4-di-O-caffeoylquinic acid, and 3- and 4-hydroxybenzoic acids); and essential oils ((*E*)-*β*-farnesene, α-zingiberene, germacrene D, (*E*)-caryophyllene, β-elemene, 2-pentylfuran, and decanal) [77]. Click or tap here to enter text.	Antimicrobial and analgesic activity. Beneficial in he treatment of rheumatic pain, possessing wound healing properties [77]. Antioxidant activity: IC_50_: 2.38 mg/mL (DPPH assay); IC_50_: 1.11 mg/mL (FRAP assay) [77].	Not reported.
*Helminthotheca echioides* (L.) Holub	Sesquiterpene lactones—guaianolides jacquinelin, 11-epi-jacquinelin, achillin, and eudesmanolide telekin; and monoterpene glucosides [78,79], as well as phenolic compounds with the most abundant luteolin and apigenin derivates [80].	Antioxidant [78] and antimicrobial [80]. Antibacterial activity: *B. cereus* (MIC 0.15 mg/mL); *S. aureus* (MIC 0.30 mg/mL); *S. typhimurium* (MIC 0.20 mg/mL) [80].	Not reported.
*Hyoseris radiata* L.	Hydroxycinnamic acids, flavonoids, megastigmane glucosides, coumarins, and lignans, together with several unsaturated fatty acids.	Diuretic [63]. Antioxidant activity: IC_50_: 2.43 mM (DPPH assay); IC_50_: 3.13 mM (ABTS assay) [81]. Anti-inflammatory activity: Inhibited COX-2 expression (50 µg/mL vs. LPS) [81]. Hypolipidemic activity: IC_50_: 39.8 μg/mL [82].	Not reported.
*Hypochaeris achyrophorus* L.	Sesquiterpenoids (8α-hydroxyhypoglabric acid and its 12,8-olide) [83].	Not reported.	Not reported.
*Hypochaeris cretensis* (L.) Bory & Chaub.	Terpenes (taraxasterol, lupeol and its acetate and Δ12-isomer, phytol, isoalantolactone, hypocretenofide, and methyl hypocretenoate) [84] and sesquiterpene lactones, namely, the rare class hypocretenolides [85].	Cytotoxic and potential to induce apoptosis [85].	Not reported.
*Hypochaeris radicata* L.	Alkaloids (nicotine, colchicine, and strychnine), phenolic compounds (isoquercetin, chlorogenic acid, kaempferol, and quercetin, coumarin), glycosides, saponins, and terpenoids (lupeol) [86].	Anti-diabetic effect. α-glucosidase inhibitory activity IC_50_: 37.6 µg/mL; α-amylase inhibitory activity IC_50_: 56.9 µg/mL; and lipase inhibitory activity IC_50_: 52.4 µg/mL [82].	Not reported.
*Inula helenium* L.	Eudesmane-type sesquiterpenes [87], phenolic acid, flavonoids, and inulin [88].	Digestive and respiratory diseases, anti-inflammatory, antioxidant. and neuroprotective [17,88]. Anti-proliferation activity as isoalantolactone on pancreatic cancer and cells: PANC-1 IC_50_: 3.75 µg/mL and SW1990 IC_50_: 3.15 µg/mL [89]. Anthelmintic, antistaphylococcal, and antibacterial [90].	Not reported.
*Lactuca serriola* L.	Essential oil with hexadecanoic and oleic acid as dominant components [91], triterpenoid compounds, fatty acids, fatty acid esters, dicarboxylic acid esters [92], sesquiterpene lactones in the latex—lactucin-type guaianolides (lactucin, lactucopicrin, 11β,13-dihydrolactucin, and 11β,13-dihydrolactucopicrin) [93].	Sedative [63,93]; treatment of headache, insomnia, nervousness, hypertension, palpitation, fever, etc.; sedative, hypnotic, diuretic, antioxidant, anesthetic, antispasmodic, anticancer, bronchodilator, and vasorelaxant effects [94]. Antibacterial activity: *S. aureus*, *P. aeruginosa*, and *C. albicans* (MIC 0.94 μL/mL); *C. parapsilosis* (MIC 0.47 μL/mL); *K. pneumoniae* (MIC 1.87 μL/mL) [91]. Analgesic, anti-inflammatory, and antioxidant properties [91,93].	Not reported.
*Lapsana communis* L.	Flavonoids (quercetin-3-O-(2″-O-rhamnosyl)rutinoside, kaempferol-3-O-(2″-O-rhamnosyl)rutinoside, quercetin-3-*O*-rutinoside, quercetin-3-O-hexoside, quercetin-3-O-(6″-O-malonyl)-glucoside, kaempferol-3-*O*-rutinoside, kaempferol-3-*O*-glucoside, and kaempferol-3-O-(6″-O-malonyl)-glucoside); phenolic acids (3-*O*-caffeoylquinic, cis-3-O-p-coumaroylquinic, trans-3-O-p-coumaroylquinic, and 4- and 5-O-caffeoylquinic acids); calcones (xanthohumol); essential oils (α-humulene, β-caryophyllene, γ- and δ-cadinene, β-farnesene, and borneol); α- and β-bitter acids (cohumulone, humulone, colupulone, lupulone [95,96], triterpene alcohols, and the fatty acids of nonsaponifiable matter [97]) and sesquiterpene lactone glycosides: crepiside E, tectoroside, lapsanoside A, lapsanoside B, and lapsanoside C in the latex of young stems [98].	Antibacterial but no cytotoxic activity [98] and volatile oils reveal powerful anti-inflammatory activity [96].	Not reported.
*Leontodon tuberosus* L.	Phenolic compounds (chlorogenic acid, 3,5-dicaffeoylquinic acid, and lignan glicosides such as 2,4,6-trihydroxyacetophenone, 2-O-β-D-glucopyranoside, and syringaresinol 4′-O-β-D-glucopyranoside) and sesquisterpenes (1,2-dehydro-3-oxocostic acid β-D-glucopyranosyl ester) [99].	Not reported.	Not reported.
*Onopordum acanthium* L.	Saponins, alkaloids, sesquiterpene lactones (4β,14-dihydro-3-dehydrozaluzanin C, zaluzanin C, and 4β,15,11β,13-tetrahydrozaluzanin C), triterpenes, sterols, nitrogen-containing compounds, phenolic compounds—lignans (serotonin derivatives, arctiin, arctigenin, and matairesinol), the neolignan nitidanin-diisovalerianate, flavonoids, phenolic acids with hydroxycinnamic acids as major compounds, coumarins, inulin, soluble sugars, proteins, and oils [100,101,102,103].	Used traditionally as bactericide, cardiotonic and hemostatic, and diuretic to treat nervousness, inflammation of the bladder, and the respiratory and urinary systems. Anti-inflammatory, analgesic, antipyretic, antiepileptic, and wound healing activities [102,104,105,106]. Antibacterial activity: *S. epidermidis*, *M. luteus* (MIC ~612 μg/mL) [104]. Hypotensive activity: IC_50_: 180–300 µM [105]. Antioxidant activity: IC_50_: ~2.6 μg/mL (DPPH assay) [105,106]. Cytotoxic activity: U-373 (glioblastoma); IC_50_: ~309 μg/mL [105,106].	Safe and non-toxic [105,107].
*Onopordum illyricum* L.	Sesquiterpene lactones, triterpenes, polyphenols—flavones and caffeoylquinic acids and their derivatives, hydroxycinnamic acid, flavonol derivatives, cynarin, 1-succinyl, 3,5-dicaffeoylquinic acid, arctiin, hispidulin, luteolin, apigenin, apigenin 4′-O-methyl ether, apigenin rutinoside, chlorogenic acid, rhamnetin, rhamnetin rutinoside, kaempferide glucoside, and sesquisterpenes (vernomelitensin, 8-(4′-hydroxymethacryloyl)-dehydromelitensin, elemacarmanin, onopordopicrin, carmanin, 8α-(5-hydroxy)-angeloylsalonitenolide, and onopordopicrin) [108,109,110,111,112,113,114].	Antioxidant, antiradical, and anticholinergic properties [110]. Anti-inflammatory activity: Reduced TNFα-induced IL-8 secretion; IC_50_: ~12 μg/mL; Reduced NF-κB activity in gastric AGS cells (IC_50_: 0.65 μM [111,113]. Anti-HIV-1: IC_50_: 8.8 μg/mL [112].	Lack of toxicity [108].
*Reichardia picroides* (L.) Roth	Polyphenols, flavonoids, and the isolated pure compound luteolin 7-O-β-D-glucoside [115].	Antioxidant, hypoglycemiant, diuretic, depurative, galactagogue and tonic agent, and anti-hemolytic protective [63,115,116]. Gastroprotective activity (in vivo). Effective dose: 500 mg/kg reduced ulceration index and increased protection percentage [116].	Toxicity assays reveal a lethal dose of chloroform extract superior to 5000 mg/kg [115].
*Scolymus grandiflorus* Desf.	Stigmasterol, γ-sitosterol, lupeol, lupeol acetate, and β-amyrin. Phytochemicals, such as 2-linoleoylglycerol, γ-sitosterol, β-amyrin, lupeol, (3α)-12-oleanen-3-yl acetate, and lupenyl acetate [94], as well as essential oil with davanone and davanol D1 and 2-hydroxy-davanone as dominant constituents [117].	Essential oil rich in davanone and davanol D-1 and 2-hydroxy-davanone may be a new source of non-toxic anticancer agents [94,117]. Antioxidant cctivity: IC_50_: 0.75 mg/mL (DPPH assay) [94]; IC_50_: 0.61 mg/mL (ABTS assay) [94]. Antibacterial activity: *S. aureus*; *C. albicans* (MIC 2.5 mg/mL); and *E. coli* (MIC 5 mg/mL) [117,118].	Not reported.
*Scorzoneroides cichoriacea* (Ten.) Greuter	Germacrane-type sesquiterpenoids (glucozaluzanin C and 15-β-D-glucopyranosyl-8-[p-(β-D-glucopyranosyloxy) phenylacetyl]-salonitenolide) [119].	No cytotoxic activity [119].	Not reported.
*Silybum marianum* L. Gaertn.	Milk thistle is mostly known for the flavonolignans silymarin, silibinin (silybin A and silybin B), isosilibinin, silychristin, silydianin, and others [120,121,122] and flavonoids (taxifolin, quercetin, etc.) [122,123], as well as phenolic acids [122].	Antioxidant activity: IC_50_: 19.2 μg/mL (DPPH assay); IC_50_: 7.2 μg/mL (ABTS assay); IC_50_: 22.2 μg/mL (CUPRAC assay); IC_50_: 24.1 μg/mL (FRAP assay) [122]. Antidiabetic effects: α-glucosidase inhibitory activity IC_50_: 18.1 µg/m; α-amylase inhibitory activity IC_50_: 26.5 µg/mL [122]. Gastrointestinal disorders—relief of dyspepsia and digestive complaints of hepatic origin [124].	Non-toxic [49].
*Sonchus arvensis* L.	Steroids (ergost-6,22-diene-3β,5α,8α-triol, ergost-5,22-diene-3β,7α-diol, stigmasterol-5-ene-3β,7α-diol, stigmasterol-7,22-diene-3β,5α,6β-triol, β-sitosterol, daucosterol, and stigmasterol-4,22-diene-6β-ol-3-one); sesquiterpenes (sonchuside-E, sonchuside-F, sonchuside-G, sonchuside-H, sonchuside I, sonchuside A, and pirciside C); and vitamin C [123,125].	Antioxidant activity: IC_50_: 341.2 μg/mL aqueous extract (DPPH assay) [126]. IC_50_: 366.6 μg/mL methanol extract (DPPH assay) [126]. Xanthine oxidase inhibitory activities: IC_50_: 81.73 μg/mL aqueous extract; IC_50_: 78.81 μg/mL methanol extract [126].	Non-toxic [127].
*Sonchus asper* (L.) Hill	Fatty acids; vitamins; phenolic acids (gallic, caffeic, coumaric, and acids); flavonoids (luteolin, rutin, and quercetin and its derivatives); sesquiterpene lactons; alkaloids; and phytic acid [128,129].	Anti-inflammatory, antimicrobial, antioxidant, antidiabetic, and cardioprotective [130].	Non-toxic [130].
*Sonchus oleraceus* L.	Sesquiterpene glycosides (glucozaluzanin C, macrocliniside A, crepidiaside A, picriside B, picriside C, and sonchusides A-D) [131]; flavonoids (luteolin, luteolin-7-O-β-D-glucoside, apigenin, kaempferol, quercetin, apigetrin, astragalin, and isoquercetin), phenolic acids, and essential oils [132].	Not reported.	Not reported.
*Taraxacum officinale* aggr.	Sesquiterpenoids (tetrahydroridentin B, taraxacolide 1-O-β-D-glucopyranoside, taraxinic acid β-D-glucopyranosyl ester, and ixerin D); steroids and triterpenoids (sitosterol, stigmasterol, campesterol, and taraxasteryl acetate); phenolic acids (quinic, caftaric, coutaric, chicoric, caffeoylquinic, and chlorogenic acids); flavonoids (luteolin, chrysoeriol, and apigenin and its derivatives); wax and latex; tocopherols; carotenoids; fibers; and minerals [133].	Diuretic [63].	Non-toxic [134] but the excessive consumption of officinale could be contra-indicated, due to a particular sesquiterpene lactone [63].
*Tragopogon dubius* Scop.	Terpenes, phenolic compounds, flavonoids, phenolic acids (including chlorogenic and rosmarinic acids), tannins, alkaloids, and carbohydrates [135,136,137].	Ethyl acetate extract—potent inhibitor of acetylcholinesterase butyrylcholinesterase, and noteworthy activity against α-glucosidase [135]. Antioxidant activity [136]: IC_50_: ~132.52 μg/mL (DPPH assay); IC_50_: ~173.87 μg/mL (ABTS assay); IC_50_: ~ 128.28 μg/mL (Superoxide assay). Cytotoxic activity [136]: A549 (lung carcinoma) IC_50_: 31.62 μg/mL.	Not reported.
*Tussilago farfara* L.	Phenolic compounds including chlorogenic and rosmarinic acids; flavonoids and flavonols (kaempferol and its 3-O-β-glucopyranoside, 3-O-α-rhamnopyranosyl(1→6)-β-glucopyranoside, and quercetin derivatives: 3-O-β-arabinopyranoside, 3-O-β-glucopyranoside, and 3-O-α-rhamnopyranosyl(1→6)-β-glucopyranoside) [135,138,139]; sesquiterpenoids (including norsesquiterpenoid—tussfarfarin A) [140,141], patchoulane, 17-pentatriacontene less m-cymene, 5-tridecene, cubebene, germacrene D, (−)-spathulenol, bisabolene epoxide, dibutyl phthalate, 2-hexadecanol, dehydro-aromadendrene, campesterol, and stigmasterol, where the quantities of the components vary between leaves and flowers [142]); pyrrolizidine alkaloids (senkirkine, senecionine and saturated otonecine-type tussilagine, and isotussilagine) [143,144,145,146]; prenylated indole alkaloids; and lignans (flower buds) [147].	Traditionally used for treating respiratory, digestive, and circulatory ailments; neuroprotective, anti-inflammatory, antioxidant, and anticancer activities [141]; and ethyl acetate extract—potent inhibitor of acetylcholinesterase and butyrylcholinesterase, as well as noteworthy activity against α-glucosidase of methanol extract [135,139]. Sesquiterpenoids isolated from the flower buds inhibit diacylglycerol acyltransferase [148].	A relatively low level of toxic pyrrolizidine alkaloids [138,145].
*Urospermum dalechampii* (L.) Scop. ex F.W. Schmidt	Terpenes (urospermal A, l1βH,13-dihydrourospermal A, loliolide, rospermal A 15-O-glucoside 6′-p-hydroxyphenylacetate, and zaluzanin C), flavonoids (naringenin, aromadendrin, and dihydroflavonol 3-O-methyltaxifolin) [149,150], and essential oils (palmitic acid, henecosane, 2-methyl-Z,Z-3,13-octadecadienol, tricosane, dill apiole, myristic acid, myristicin, 2-pentadenanone-6,10,14-trimethyl, lauric acid, elemicin, isobutyl phlatate, caryophyllene oxide, epicubenol-1, β-guaiene, α-bisabolol, and 3,7-dimethyl-octa-1,6-diene) [151].	Analgesic activity (in vivo): 200 mg/kg 68.4% inhibition of writhing response. Anti-inflammatory (carrageenan-induced pleurisy): 200 mg/kg reduced neutrophil migration [152].	No signs of acute toxicity in vivo (test dose of 2000 mg/kg) [152].

Some of the wild edible plants from the family Asteraceae that are well known and used in traditional and official medicine have been vastly studied phytochemically and pharmacologically. Such recognized medicinal plants are *Silybum marianum*, *Artemisia vulgaris*, *Taraxacum officinale*, and *Tussilago farfara*, as well as *Cichorium intybus.* The last one is also industrially utilized in gastronomy as a coffee substitute, food or drink additive, and some other uses (Table 2). Additionally, they are introduced in culture for medicinal purposes or other industrial applications. Chicory byproducts (ranging from leaves to roots and pulp) offer valuable applications in livestock feed, food, pharmaceuticals, cosmetics, biorefineries, and green chemistry, making them key resources for sustainable agriculture and circular economy strategies [153]. Additionally, good practices illustrating how sustainable use can be achieved through cultivation are reported in Spain for *Scolymus hispanicus* L. [7].

Although the endemic plant *Carlina sicula* is popular as a green vegetable [154,155] it is scarcely studied regarding its bioactive compounds, pharmacological activity, and toxicity (Table 2, ref. [40]. The same is true for *Centaurea napifolia*, *Leontodon tuberosus*, *Scorzoneroides cichoriacea*, *Reichardia picroides*, etc. (Table 2), although these plants have a wider range of distribution (Table 1).

The group of edible species with a wide distribution and numerous populations, such as *Carduus nutans*, *Centaurea cyanus*, *Lactuca serriola*, *Lapsana communis*, *Sonchus oleraceus*, *Sonchus asper*, *Onopordum acanthium*, *Onopordum illyricum*, *Tragopogon dubius*, etc., is also not sufficiently studied for their pharmacological effects and little is known about their bioactive compounds. This group deserves further attention, because some species might be disqualified due to toxicity. However, other species that are widespread, some of them ruderals and considered weeds, might appear good candidates for food or extensive pharmaceutical use and even introduction in culture.

The main groups of compounds reported in the Asteraceae species traditionally used as food, as well as their biological activities and toxicity, are summarized in Table 3, Table 4 and Table 5. The main limitation of the existing literature is the lack of consistency among available studies regarding the phytochemical data and toxicity. The details for each species can be traced in Table 2. It is not surprising that the species widely recognized for their medicinal uses such as *Artemisia vulgaris*, *Cichorium intybus*, *Inula helenium*, *Silybum marianum*, *Taraxacum officinale*, *Tussilago farfara*, etc. have been studied more extensively than other species.

### 3.3. Toxicity Assays

The toxicological studies related to the edible plants listed here are scarce in general (Table 2). Detailed toxicity tests of the plants that contain sesquiterpene lactones are not sufficient. The same is true for the toxicity tests of the plants that contain alkaloids.

Furthermore, the method of consumption is important. Numerous studies show that processing the plant material affects the bioactive compounds. For example, fresh *Hyoseris radiata* and *Hypochaeris radicata* have hypolipidemic and hypoglycemic activities, and blanching reduces them [82]. Therefore, it is recommended to reuse the blanching water in food preparation since it is a good source of bioactive compounds [82]. Cooking temperature affects alkaloid degradation, but the results are inconsistent and depend on the specific alkaloid, food matrix, and processing conditions, with some alkaloids showing significant degradation and others remaining relatively stable [156,157]. Sesquiterpene lactones degrade with heat processing, though degradation is compound- and temperature-dependent; for example, some compounds increase at 60–80 °C while others decrease, and degradation is more pronounced at higher temperatures [158,159]. Therefore, detailed studies are required for each species.

## 4. Conclusions

The ethnobotanical data reveal a substantial number of wild edible plants belonging to the family Asteraceae. This study provides a systematic comparison of traditional edible plants from the family Asteraceae between the Mediterranean and Balkan regions. The principal metabolite classes identified include flavonoids, phenolic acids, lignans, and sesquiterpene lactones, all of which are linked to antioxidant, anti-inflammatory, hypolipidemic, and anticancer properties. Taken together, these findings highlight the promising potential of such species as future health-promoting foods. At the same time, specific taxa within the investigated group were found to contain alkaloids that warrant toxicological attention. Some species within the studied group contain alkaloids that may pose toxicity risks. *T. farfara* is known to accumulate pyrrolizidine alkaloids, which are associated with liver damage and potential genotoxic effects, highlighting the importance of thorough toxicological evaluations before recommending these plants for consumption. This study also demonstrates how common culinary processes, such as boiling and blanching, significantly impact the concentration, stability, and safety profile of these bioactive compounds. Overall, the research supports the promotion of wild Asteraceae species as valuable, sustainable, nutritional, and nutraceutical resources. This approach aligns with efforts to preserve biodiversity and maintain traditional Mediterranean and Balkan food practices, integrating both ecological and cultural sustainability. One of the main limitations of the published literature is the lack of consistency across the available studies. The present study represents an initial step toward identifying current knowledge gaps and highlighting priorities for future research. For many of these plants, information on toxicity is lacking, so they may be classified as “understudied” in this regard.

## Figures and Tables

**Table 1:** Wild plants from family Asteraceae traditionally used for food in Bulgaria and Sicily.

Taxon	Distribution in the Study Sites	Used in the Study Sites	Used Parts and Modes of Application as Food
*Artemisia vulgaris* L.	BG, Sicily	BG	Leaves, pastries.
*Bellis perennis* L.	BG, Sicily	BG Sicily	Leaves, raw salad, soup. Tender leaves of the basal rosette, raw in salads, simply stewed or as an ingredient in soups.
*Carduus nutans* L.	BG, Sicily	BG	Sprouts, young herbage spines removed, stew.
*Carlina gummifera* (L.) Less. (Syn. *Atractylis gummifera* L.)	Sicily	Sicily	Fleshy receptacle of the capitula (inflorescences), raw or stewed.
*Carlina sicula* Ten.	Sicily	Sicily	Tender stems, stewed, seasoned with oil and lemon, or fried with eggs.
*Centaurea calcitrapa* L.	BG, Sicily	Sicily	Tender leaves of the basal rosette, boiled and simply seasoned with salt and olive oil.
*Centaurea cyanus* L.	BG, Sicily	BG	Young herbage.
*Centaurea napifolia* L.	Sicily	Sicily	Boiled basal leaves, alone or with other wild greens, eaten with olive oil, salt, and lemon juice.
*Cichorium intybus* L.	BG, Sicily	BG Sicily	Roots, leaves, shoots, raw salad, soup, coffee surrogate/beverage. Basal rosette leaves, raw in salads or stewed.
*Crepis leontodontoides* All.	Sicily	Sicily	Basal rosette leaves boiled and simply seasoned with salt and olive oil.
*Crepis sancta* (L.) Bornm.	BG, Sicily	BG	Young herbage.
*Crepis vesicaria* L. subsp. *vesicaria*	Sicily	Sicily	Basal rosette leaves, raw in salads or simply stewed or as an ingredient in soups and omelets.
*Cynara cardunculus* L.	Sicily	Sicily	Tender leaves and parts of the stem, stewed or fried in batter; capitula (inflorescences) for preserves in olive oil or vinegar.
*Doronicum orientale* Hoffm.	BG, Sicily	BG	Leaves.
*Helminthotheca echioides* (L.) Holub (Syn. *Picris echioides* L.)	BG, Sicily	Sicily	Basal leaves, stewed with other vegetables, then sautéed and seasoned with garlic, olive oil, chilli, and lemon juice.
*Hyoseris radiata* L.	Sicily	Sicily	Basal leaves, boiled and simply seasoned with salt and olive oil, or as an ingredient in soups.
*Hypochaeris achyrophorus* L.	Sicily	Sicily	Basal rosette leaves, boiled and simply seasoned with salt and olive oil.
*Hypochaeris cretensis* (L.) Bory & Chaub.	BG, Sicily	Sicily	Basal rosette leaves, boiled and simply seasoned with salt and olive oil.
*Hypochaeris radicata* L.	BG, Sicily	Sicily	Basal rosette leaves, raw in salads, blanched or stewed as an ingredient in soups; omelets.
*Inula helenium* L.	BG	BG	Roots, soup, beverage.
*Lactuca serriola* L.	BG, Sicily	BG	Sprouts, young leaves, raw salad, soup.
*Lapsana communis* L.	BG, Sicily	BG	Leaves.
*Leontodon tuberosus* L.	BG, Sicily	Sicily	Basal rosette leaves boiled and simply seasoned with salt and olive oil.
*Onopordum acanthium* L.	BG	BG	Roots, leaves, shoots (less than 20 cm tall), raw salad, soup, coffee surrogate/beverage.
*Onopordum illyricum* L.	BG, Sicily	Sicily	Leaf veins and the basal part of the tuft of leaves and roots stewed or fried in batter.
*Reichardia picroides* (L.) Roth	BG, Sicily	Sicily	Basal rosette leaves, raw in salads, stewed or as an ingredient in soups.
*Scolymus grandiflorus* Desf.	Sicily	Sicily	Tufts of basal leaves simply stewed or fried in batter.
*Scorzoneroides cichoriacea* (Ten.) Greuter [Syn. *Leontodon cichoraceus* (Ten.) Sanguin.]	BG, Sicily	Sicily	Basal rosette leaves boiled and simply seasoned with salt and olive oil.
*Silybum marianum* (L.) Gaertn.	BG, Sicily	BG Sicily	Leaves, sprouts, young anthodia, salad, soup, stewed. Tender shoots, raw in salads or as an ingredient in soups; capitula (inflorescences) receptacles stewed.
*Sonchus arvensis* L.	BG	BG	Leaves, raw salad, soup.
*Sonchus asper* (L.) Hill	BG, Sicily	BG Sicily	Leaves, raw salad, soup. Basal leaves, raw in salads or boiled and simply seasoned with salt and olive oil.
*Sonchus oleraceus* L.	BG, Sicily	BG Sicily	Leaves, raw salad, soup. Whole young plant or tender shoots of the adult stem, raw in salads or boiled and simply seasoned with salt and olive oil.
*Taraxacum officinale* aggr.	BG, Sicily	BG	Leaves, young anthodia, salad, marinated anthodia.
*Tragopogon dubius* Scop.	BG	BG	Leaves, raw salad, soup, stew.
*Tussilago farfara* L.	BG, Sicily	BG	Young leaves, sprouts, anthodia, salad blanched, soup, pastry.
*Urospermum dalechampii* (L.) Scop. ex F.W. Schmidt (Syn. *Tragopogon dalechampii* L.)	Sicily	Sicily	Basal rosette leaves, boiled and simply seasoned with salt and olive oil.

**Table 3:** Polyphenols in selected Asteraceae species.

Subclass/Main Compounds	Plant Species	Biological Activities	Toxicity
**Flavonoids:** apigenin, luteolin, kaempferol, quercetin, rutin, chrysoeriol, vitexin, hesperidin, naringenin, catechin, galangin, and isorhamnetin [28,30,41],	*Artemisia vulgaris*, *Bellis perennis*, *Carduus nutans*, *Centaurea calcitrapa*, *Centaurea cyanus*, *Centaurea napifolia*, *Cichorium intybus*, *Crepis vesicaria*, *Crepis sancta*, *Doronicum orientale*, *Helminthotheca echioides*, *Hypochaeris radicata*, *Inula helenium*, *Lapsana communis*, *Onopordum acanthium*, *Onopordum illyricum*, *Reichardia picroides*, *Silybum marianum*, *Sonchus asper*, *Sonchus oleraceus*, *Taraxacum officinale*, *Tragopogon dubius*, *Tussilago farfara*, and *Urospermum dalechampii*	Antioxidant activity: IC_50_: 11.4 µg/mL (DPPH assay) [26]; IC_50_: 125 mg/mL (NO assay) [26]. Cytotoxic activity: MCF-7 (breast cancer) IC_50_: 190 ng/mL; HeLa (cervical cancer) IC_50_: 284 ng/mL; A7R5 (smooth muscle cells) IC_50_: 382 ng/mL; 293T (transformed kidney cells) IC_50_: 317 ng/mL; A549 (lung carcinoma) IC_50_: 778 ng/mL [26]. Antispasmodic/ antinociceptive activity (in vivo). Effective doses: 500 mg/kg and 1000 mg/kg. Anti-inflammatory/hypolipidemic/hepatoprotective (in vivo). Effective dose: 100–600 mg/kg [26]. Estrogenic, antimicrobial, antiallergic, antimalarial, and gastrointestinal digestion stimulation [26]. Anxiolytic/antidepressant-like effects (in vivo). Effective doses: 50, 100, and 150 mg/kg [31].	Generally non-toxic; LD50 5000 mg/kg for *Reichardia picroides* [115]
**Phenolic acids:** chlorogenic acid, caffeic acid, rosmarinic acid, chicoric acid, dicaffeoylquinic acids, cynarin, ferulic acid, p-coumaric acid, and gallic acid [30,34,41]	*Artemisia vulgaris*, *Bellis perennis*, *Carduus nutans*, *Centaurea calcitrapa*, *Centaurea cyanus*, *Cichorium intybus*, *Crepis vesicaria*, *Crepis sancta*, *Cynara cardunculus*, *Doronicum orientale*, *Helminthotheca echioides*, *Hyoseris radiata*, *Leontodon tuberosus*, *Onopordum acanthium*, *Onopordum illyricum*, *Sonchus asper*, *Sonchus oleraceus*, *Taraxacum officinale*, *Tragopogon dubius*, *Tussilago farfara*, and *Urospermum dalechampii*	Antioxidant, anti-inflammatory, hepatoprotective, and antimicrobial.	Non-toxic at tested concentrations; non-mutagenic

**Table 4:** Alkaloids in selected Asteraceae species.

Subclass/Main Compounds	Plant Species	Biological Activities	Toxicity
**Generic and specific alkaloids:** siculamide (unique alkaloid from *Carlina sicula*), nicotine, colchicine, strychnine, pyrrolizidine alkaloids (senkirkine, senecionine, tussilagine, and isotussilagine), and prenylated indole alkaloids [40,135]	*Carlina sicula* [siculamide], *Centaurea cyanus*, *Hypochaeris radicata* [nicotine, colchicine, and strychnine], *Onopordum acanthium*, *Sonchus asper*, *Tragopogon dubius*, and *Tussilago farfara* [pyrrolizidine alkaloids and prenylated indoles]	Anti-inflammatory, antidiabetic, antitumor, acetylcholinesterase inhibition, and glucose uptake stimulation (siculamide and dihydrocnicin) [40].	Very low toxicity; *Tussilago farfara* contains low levels of toxic pyrrolizidines; and *Onopordum* is safe and non-toxic [105]

**Table 5:** Guaianolides, elemanolides, eudesmanolides, germacranolides, terpenes, and others in selected Asteraceae species.

Subclass/Main Compounds	Plant Species	Biological Activities	Toxicity
**Guaianolides:** lactucin, lactucopicrin, 11*β*,13-dihydrolactucin, jacquinelin, 8-deoxylactucin, and dihydrolactucopicrin. **Elemanolides, eudesmanolides, germacranolides:** cnicin, 4′-O-acetylcnicin, melitensin, dehydromelitensin, zaluzanin C, and vernomelitensin. **Hypocretenolides** (a rare class). **Others:** crepiside E, tectoroside, lapsanoside A–C, ixerin D, and urospermals [51,57,68]	*Artemisia vulgaris* [psilostachyin, psilostachyin C, vulgarin, and artemisinin], *Carlina gummifera*, *Carlina sicula*, *Centaurea calcitrapa*, *Centaurea cyanus*, *Centaurea napifolia*, *Cichorium intybus*, *Crepis vesicaria*, *Crepis sancta*, *Crepis leontodontoides*, *Helminthotheca echioides*, *Hypochaeris cretensis*, *Lactuca serriola*, *Lapsana communis*, *Onopordum acanthium*, *Onopordum illyricum*, *Scorzoneroides cichoriacea*, *Sonchus asper*, *Sonchus oleraceus*, *Taraxacum officinale*, and *Urospermum dalechampii*	Antimicrobial, antiviral, and laxative [57]. Antioxidant activity: IC_50_: 11.4 µg/mL (DPPH assay) [26]; IC_50_: 125 mg/mL (NO assay) [26]. Cytotoxic activity: MCF-7 (breast cancer) IC_50_: 190 ng/mL; HeLa (cervical cancer) IC_50_: 284 ng/mL; A7R5 (smooth muscle cells) IC_50_: 382 ng/mL; 293T (transformed kidney cells) IC_50_: 317 ng/mL; A549 (lung carcinoma) IC_50_: 778 ng/mL [26]. Antispasmodic/ antinociceptive activity (in vivo). Effective doses: 500 mg/kg and 1000 mg/kg. Anti-inflammatory/hypolipidemic/hepatoprotective (in vivo). Effective dose: 100–600 mg/kg [26]. Estrogenic, antimicrobial, antiallergic, antimalarial, and gastrointestinal digestion stimulation [26].	Variable: *Carlina gummifera* is toxic (atractyloside and carboxyatractyloside inhibit oxidative phosphorylation) [39]; *Cichorium intybus* is non-toxic and non-mutagenic up to 1000 mg/kg/day [58]
**Triterpenes:** taraxasterol, lupeol, sitosterol, stigmasterol, and campesterol. **Sesquiterpenoids:** tussfarfarin A and eudesmane-type. **Coumarins:** esculin, umbelliferone, and scopoletin. **Essential oils:** 1,8-cineole, sabinene, camphor, camphene, caryophyllene oxide, *α*-thujone, and *β*-thujone [23,24,25]	*Artemisia vulgaris*, *Bellis perennis*, *Carlina gummifera* [carlina oxide and 13-methoxy-carlina oxide], *Centaurea calcitrapa*, *Centaurea napifolia*, *Doronicum orientale*, *Hypochaeris cretensis*, *Hypochaeris radicata*, *Inula helenium*, *Lapsana communis*, *Scolymus grandiflorus*, *Sonchus arvensis*, *Taraxacum officinale*, *Tussilago farfara*, and *Urospermum dalechampii*	Antitumoral, antioxidant, wound healing, and anticancer [117,152]. Antibacterial activity: *S. aureus*, *C. albicans* (MIC 2.5 mg/mL); and *E. coli* (MIC 5 mg/mL) [117]. Analgesic activity (in vivo): 200 mg/kg 68.4% inhibition of writhing response. Anti-inflammatory (carrageenan-induced pleurisy). 200 mg/kg reduced neutrophil migration [152].	*Artemisia vulgaris* is toxic after prolonged use [26]; *Carlina gummifera* is toxic; others are generally non-toxic [117,152]

## Data Availability

No new data were created or analyzed in this study. Data sharing is not applicable to this article.
